# 

**Published:** 1884-04

**Authors:** 


CONTENTS.

| | PAGE. |
|---|---|
| Sources of Disease | 59 |
| Tact | 60 |
| Corpulence | 61 |
| Constipation | 62 |
| Entire Wheat Flour | 64 |
| Mineral Waters | 65 |
| Bright's Disease | 66 |
| Feminine Fastness | 67 |
| Beef Tea *versus* True Food | 68 |
| Health of Cities | 70 |
| Sunlight and Health | 71 |
| A Cheerful Home | 72 |
| Tired Mothers | 72 |
| Observations on Malaria | 73 |
| Pure Air | 74 |
| Effects of a Warm Foot-Bath | 74 |
| Flowers in the Sick Room | 75 |
| Shortsightedness | 75 |
| Limitations of Mind | 76 |
| Destroying a Treasure | 77 |
| Sea Voyaging | 78 |

